# Brain connectivity during encoding and retrieval of spatial information: individual differences in navigation skills

**DOI:** 10.1007/s40708-017-0066-6

**Published:** 2017-05-16

**Authors:** Greeshma Sharma, Klaus Gramann, Sushil Chandra, Vijander Singh, Alok Prakash Mittal

**Affiliations:** 1 Biomedical Engineering Department, INMAS, DRDO, Delhi, 110054 India; 2 Biological Psychology and Neuroergonomics, Institute of Technology, University of Berlin, 10587 Berlin, Germany; 3 Instrumentation and Control Engineering Department, NSIT, Dwarka, Delhi 110078 India

**Keywords:** Spatial navigation, Spatial memory, Brain connectivity, Reference frame proclivity, Graph theory

## Abstract

Emerging evidence suggests that the variations in the ability to navigate through any real or virtual environment are accompanied by distinct underlying cortical activations in multiple regions of the brain. These activations may appear due to the use of different frame of reference (FOR) for representing an environment. The present study investigated the brain dynamics in the good and bad navigators using Graph Theoretical analysis applied to low-density electroencephalography (EEG) data. Individual navigation skills were rated according to the performance in a virtual reality (VR)-based navigation task and the effect of navigator's proclivity towards a particular FOR on the navigation performance was explored. Participants were introduced to a novel virtual environment that they learned from a first-person or an aerial perspective and were subsequently assessed on the basis of efficiency with which they learnt and recalled. The graph theoretical parameters, path length (PL), global efficiency (GE), and clustering coefficient (CC) were computed for the functional connectivity network in the theta and alpha frequency bands. During acquisition of the spatial information, good navigators were distinguished by a lower degree of dispersion in the functional connectivity compared to the bad navigators. Within the groups of good and bad navigators, better performers were characterised by the formation of multiple hubs at various sites and the percentage of connectivity or small world index. The proclivity towards a specific FOR during exploration of a new environment was not found to have any bearing on the spatial learning. These findings may have wider implications for how the functional connectivity in the good and bad navigators differs during spatial information acquisition and retrieval in the domains of rescue operations and defence systems.

## Introduction

Accurate spatial representations of our surroundings are essential for successful orientation within the environment. Spatial representation entails information about the spatial arrangements of entities based on an egocentric or an allocentric frame of reference (FOR) [1, 2]. On the basis of allocentric or/and egocentric frame of reference, a cognitive map is constructed for any given environment entailing information on metric relationships such as distance and relative position of the landmarks (equivalent to survey knowledge acquisition) [3]. Construction of a map-like representation is based on an individual’s egocentric experience through the senses (vision, kinesthesis, and others) that has to be translated into an allocentric frame of reference [4]. This translation takes place at different time scales and to different degrees of accuracy but leads to the parallel existence and use of several egocentric and allocentric frames of reference [5, 6]. The individual choice of a specific spatial frame of reference can be influenced by variables such as the context of the task, experience, time, and the goal of the journey. Of particular relevance for the present study is to understand how retrieval of spatial information is influenced when the method of acquisition is the first-person perspective followed by an aerial perspective. Torok et al. [7] demonstrated a preference for an egocentric FOR when an environment was explored from a first-person perspective as compared to the preference to use an allocentric FOR when participants learnt an environment from an aerial perspective (map).

Evolution of the mechanism of memory stems from the mechanism of navigation [8]. This extends support to the hypothesis of a phylogenetic continuity and a direct relationship between the navigation and memory processes. Within this model, the hippocampus is considered as a central hub for the shared neural mechanisms that are recruited during navigation and declarative memories (episodic and semantic memory). The dichotomy of declarative memories is distinguishable like the two forms of the spatial frame of reference. Expanding on this idea, allocentric representation and semantic memory on one hand and egocentric representation and episodic memory on the other, support a common network for memory and navigation. Along the same lines, the integration of multiple routes into a cognitive map might be related to the integration of episodic memory contents with a semantic network [9]. In addition, the series of events encoded as spatial memory can be represented with the full support of episodic memory. All these shreds of evidence reflect the interwoven networks of navigation and memory [10]. This is the focal point of our study, as we have compared good and bad navigators with respect to their retrieval of spatial knowledge (memory and navigation).

The temporal dynamics of the brain activity during spatial navigation and memory can be investigated with high temporal resolution using electroencephalography (EEG). Cognitive processes underlying memory and navigation are realised through neural activity in a distributed cortical network which can be recorded by EEG. Coherence measures of these oscillations are estimators of the synchrony between two or more continuous time series of brain activity. The instantaneous phase of a signal can be estimated by using the analytical concept of the Hilbert Transform. The quantification of phase synchronisation is crucial because it might serve as a mediator for large-scale cognitive integration and the coordination of different task relevant to neural assemblies [11]. The functional significance of phase synchronisation lies in the fact that it enables the engagement of target neurons and facilitation of inter-regional interaction with the appropriate phase differences while simultaneously suppressing out of phase neural inputs [12].

Most of the work on phase synchronisation and memory has been focused on theta (4–7 Hz) and alpha (8–13 Hz) frequency bands because these bands support long-distance cortical interactions [13, 14]. Also, Cognitive functions are often transiently coupled by theta and alpha band phase synchronisation [17]. The theta band is associated with memory maintenance [15] and the gating mechanism during information processing. In addition, activity in this band increases with task difficulty [13]. The dual function of theta oscillations is realised as synaptic plasticity and the segregation of cell assemblies in the available phase space [16]. The theta time compression mechanism restrains the representation of memory and space [16]. The alpha band interactions are the predominant characteristics of planning, perception, as well as working memory function [18]. It is imperative to conceive alpha oscillations in local and large-scale cortical network from the perspectives of two alternative hypotheses: the inhibition hypothesis and the active processing hypothesis. The inhibition hypothesis states that alpha increases in task-irrelevant areas whereas it decreases in task-related areas. The active processing hypothesis postulates that alpha oscillation plays a role in task-relevant regions. Although both hypotheses are contradictory in their fundamentals, but they are necessary to maintain the default mode network, as well as higher cognitive function [18]. Due to the central role of memory in the navigation, we investigated the theta and alpha band during a virtual navigation task. Several studies demonstrated a pronounced role of the theta and alpha band during navigation [19–21]. To gain insights into the functional connectivity during navigation, we chose the alpha band for further exploration, as it follows active and inhibition hypotheses which sustain equilibrium. This, in turn, strikes a balance between functional segregation and functional integration in terms of efficient connectivity. It reflects in the functional connectivity where harmony between opposing demands of functional integration and function segregation is maintained for economical networking.

In the present study, we investigated the brain dynamics in participants with good and bad navigation skills. Additionally, we analysed the brain connectivity changes in groups of good and bad navigators during encoding of an aerial view (map) of the environment which would encourage them to build survey knowledge. Furthermore, the acquired survey knowledge was compared to the brain dynamics during exploration of the identical environment from a first-person perspective and subsequent retrieval of a novel virtual environment. Our study addressed four core questions: (1) whether there would be changes in the brain connectivity for the encoding and the retrieval of spatial information; (2) whether the brain dynamics can be compared for navigators with good navigation skills and navigators with less pronounced navigation skills; (3) whether the proclivity to use a specific FOR plays a role in shaping navigation performance and the accompanying brain dynamics, and finally; (4) whether variation in the navigation skills affect encoding of the spatial information in memory.

## Methods

### Participants

Fifty young and healthy male participants (mean age 23.5 years, range 19–28 years) were chosen for the study. All participants, except one, were right handed. Only male participants were chosen to avoid gender biases, as it has been found in the earlier study [22] that males outperform females in specific spatial tasks. All participants were from the Netaji Subhas Institute of Technology (NSIT) and the Thapar University. Also, a written informed consent was obtained from them.

### Experimental design and procedure

**Fig. 1:**
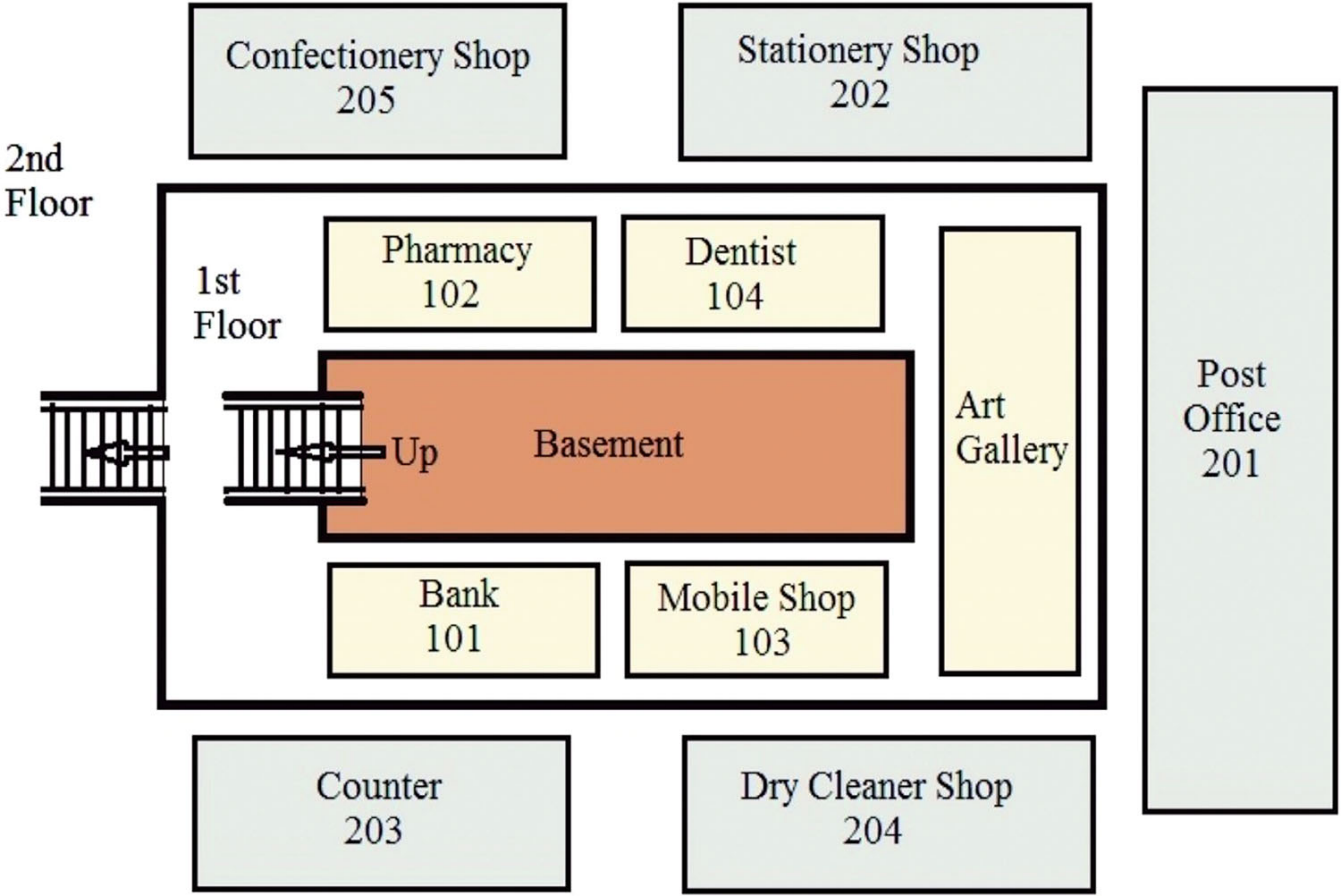
Map of the virtual environment used in this experiment

All participants underwent the *Online Reference Frame Proclivity Test* [6] which divided them on the basis of the preferred use of an egocentric or an allocentric frame of reference in a virtual path integration task. In this task, participants navigated through a star field environment, with one straight start segment, a single turn, and one straight final segment. At the end of the passage, participants selected one out of four homing vectors indicating the starting position of the passage. There were four alternative homing arrows that pointed back up, back down, back left, and back right. After a passage with a turn to the right, the participant with egocentric proclivity was found to select the homing arrow pointing back and to the left, while the participant with allocentric proclivity was found to select the homing arrow pointing back and to the right. Responses indicating left or right after a turn in the vertical direction (up or down) were considered incorrect. The same was found to be true for responses indicating up or down after heading changes in the horizontal plane. Following this, participants underwent a *virtual navigation task* designed in Unity 5. They were shown a map of a shopping complex having ten shops spread over two floors (Fig. 1). The participants were asked to remember the map of the shopping complex prior to navigating through the environment in virtual reality. The time taken to memorise the map was self-paced. This phase was termed as the encoding (Enc) phase. Once the participants had memorised the map, they entered the virtual environment i.e. shopping complex and were asked to complete a journey through the virtual environment from and back to the starting position. This phase was called the navigation (Nav) phase. In this phase, participants moved in shopping complex using mouse and keyboard while sitting calmly on a chair. They were instructed to visit each shop in the minimum time. Based on their performance, participants were classified as the ‘good navigators’ and ‘bad navigators’. The performance was evaluated on the basis of time taken to learn the map (Panning Time), the time taken to complete the navigation task (Navigation Time), the total number of shops visited (Correct Scores), the total number of shops omitted, and the total number of shops visited twice (the last two fields indicate errors (Error)). Enc and Nav phase constituted the spatial acquisition stage for the virtual navigation task in two perspectives. The participants were subsequently asked to draw a map of the environment on the paper. This time period was defined as the retrieval (Ret) phase. This phase was important to assess spatial memory for the survey knowledge (constructed via a aerial view) and route knowledge (constructed via the first-person perspective). EEG data was recorded continuously for each subject during all the three phases (Enc, Nav, and Ret).

These two tasks, *Online Reference Frame Proclivity Test* and *virtual navigation task*, were shown on 15-inch Lenovo laptop. Performance scores were saved in the text file for virtual navigation task which were further analysed for the classification of participants. Total 30 min were spent while performing the aforementioned tasks. One practice trial was presented before the beginning of the *virtual navigation task* to get acquainted with the virtual environment.

### EEG data acquisition and analysis

EEG was recorded using a EMOTIV EPOC system with 14 electrodes placed according to the extended 10% system (AF3, F7, F3, FC5, T7, P7, O1, O2, P8, T8, FC6,F4, F8, AF4). Two reference electrodes were placed at the P3/P4. Electrode impedance was kept below 5 kΩ. The data was recorded with a sampling frequency of 128 Hz. In addition, a notch filter was applied to filter out 50 Hz power line interference. Muscular, line noise, and eye blink artifacts were removed using independent component analysis (ICA) in EEGLAB toolbox [23].

The EEG system is usually designed for brain–computer interface, but several recent studies have used it for the basic research [24, 25]. The experiment was conducted in a noise-free and dimly-lit room. Three segments (prior to the task, just after cue presentation, and middle of the task) of one second each was selected for further analysis. The filtered data (after removal of artifacts) were decomposed and reconstructed using Discrete Wavelet Transform (DWT) to extract theta (4–7 Hz) and alpha (8–13 Hz) bands. Phase synchronisation between pairs of electrodes was determined by computing their phase locking value using Hilbert Transform [26]. Phase synchronisation, network measures (path length, clustering coefficient, global efficiency, degree node, and network density), and small world index were computed using brain connectivity toolbox (brain-connectivity-toolbox.net) in MATLAB [27]. Graph Theoretical analysis was based on a complete 14 × 14 matrix of the selected frequency bands.

To assess functional connectivity during the encoding and retrieval of spatial information in a virtual navigation task, the ‘Graph Theoretical analysis’ has been applied to the recorded EEG data. Functional connectivity patterns depict the arrangement of ‘*m*’ different brain areas in the form of a network. Graphs are characterised by indices such as clustering coefficient (CC), path length (PL), and global efficiency (GE). These indices describe the network as either regular (high CC and high PL) or random (short CC and short PL). Small world (SW) networks are formed with high CC as in regular networks and PL equivalent to that of random networks, with a probability *p* between 0 and 1. SW networks realise the optimal balance of functional integration and segregation. Centrality metrics such as degree and betweenness are used to identify the hubs within the network. The mean network degree is most commonly used as a measure of density or the total ‘wiring cost’ of the network. Network topology and connectivity have been hypothesised to relate to behavioural and cognitive performance in psychiatric brain disorders [28] and in resting brain condition [29]. A graph theoretical network analysis was carried out to evaluate small world networks, in which connectivity was determined by all pair-wise combinations of channels resulting in 14 × 14 connectivity matrices of phase synchronisation. Analysis of the small world network requires the degree and rewire parameters (probability of connection at every node in the network). The rewire parameter, ‘*n*’, was taken as 14 and calculated as log(*n*)/*n*. The small world network computational model was based on the rewire parameter and degree values of the 14 node connectivity and adjacency matrix for alpha band. The threshold was kept between 0.01 and 0.05, and the complete analysis was repeated throughout this range. An edge was assumed to exist if the value of phase synchronisation between a pair of channels exceeded the value of this threshold. The small world network computation gave a sparse matrix as an output on which small world characteristics were computed, namely path length (Lp) (minimum number of edges that must be traversed to get from one node to another)and clustering coefficient (Cp) (cliquishness of a local neighbourhood based on number of connections). When the clustering coefficient was high and the path length was short, a small world existed in the network. For the specific purpose of this study, signals were filtered to analyse the phase synchronisation in the theta and alpha frequency bands. Subsequently, Cp, Lp, Cr (clustering coefficient of a random network), as well as Lr (Path length of a random network) were compared with their ratios (Cp/Cr and Lp/Lr) to get small world indices (SWi). Random graphs were generated from the experimentally obtained graphs by a constrained shuffle of the vertices keeping both the number of vertices and the degree of distribution.

### Statistical analysis

The study was based on comparison-group randomised experimental design. Based on the performance matrix, ‘K-means’ Clustering method (centre = 2) was applied to classify participants as the good or bad navigators. This technique was also applied to the recall score to distinguish the best performers from the worst performers within a group. Two-way ANOVA and one-way ANOVA were employed to observe effect of FOR on response latency and performance matrix, respectively. Similarly, one-way ANOVA was applied on graph indices, PL,GE, and CC, for computing differences between the good and bad navigators. For each phase, scatter plots of recall scores were plotted with network density and SWi to evaluate ramification of individual differences.

## Results

Participants were classified as those with an egocentric strategy or allocentric strategy depending on the used corresponding FOR in at least 19 out of 24 trials (75%) [30, 31]. Subsequently, our sample size reduced from 50 to 28 participants (egocentric strategy = 21, allocentric strategy = 7). On the other hand, on the basis of performance matrix (performance scores obtained during Nav phase), further 28 participants were classified into eleven good navigators (only 1 using an allocentric strategy) and seventeen bad navigators (6 in allocentric strategy and 12 in egocentric strategy). The performance matrix was computed on the ground of following cut-off values for each variable: Correct Scores > 8, Error < 2, Navigation Time ≤ 170 s and Planning Time < 100 s.

The remainder of the result section is organised as follows: first, descriptive subsets of behavioural outcome is presented. These subsets were chosen to link spatial memory with the navigation performance in the good and bad navigators. In addition, effect of reference frame proclivity is evaluated with the performance. Second, Graph Theoretical analysis is summarised to provide a comprehensive overview of the theta and alpha bands in the three phases. Finally, the pattern of hubs activation in the cortical areas for navigators having degree of navigation skills is projected. Hubs were identified on the basis of node degree that was equal to the number of edges maintained by each node.

### Behavioural data analysis

Recall scores gathered from drawing the sketch map of the traversed route was further evaluated to measure extent of the spatial learning in the participants. Subsequently, on the basis of recall scores, participants were classified as the best and worst performers within the group. The drawn sketch map allowed for quantifying the recall of spatial relations with the number of recalled shops (*R*1), the number of order breaks (*R*2), and the percentage of completeness (*R*3) as dependent measures. The final score resulted from the agglomeration of the three components with weights of 1, −1 and 1, respectively. The negative weight associated with *R*2 reflected a penalty for each instance of the following errors: E1: If the inner and outer peripheries were not conserved or all the elements stacked in a single row; E2: If the vertical and horizontal relationships between elements were not maintained; E3: Omission of the cornered shops which was either the Art Gallery or Post Office; E4: Addition of new elements that were not present in the original map. *R*3 considered the block diagram of the spatial layout of the building, wherein the relative positions of all the shops in the building were to be maintained. Only 25% of the good navigators demonstrated an astute performance, whereas 45% in the bad navigators exhibited best performance in the retrieval phase. This might suggests that it cannot be presumed that a good navigator would also perform well in terms of memory and the two variables (recall scores and performance scores) can not be linearly correlated.

Considering online proclivity test, response latency was computed in the two categories: axis (yaw and pitch) and response type/strategy (egocentric, allocentric, and incorrect). For statistical analysis, we excluded ‘incorrect’ response type from online proclivity test. For analysis, first, a two-way ANOVA with factor axis and response type was calculated to root out differences in the response latency. The analysis revealed non-significant effect of factor axis, *F*(1, 210) = 2.8, *p* > 0.05, and factor response type, *F*(1, 210) = 3.545, *p* > 0.05. Second, one-way ANOVA was applied to see interaction effect of response latency on the navigation performance. The results showed that that neither of the two FORs introduced any bias, positive or negative, to the aggregate performance measure, *F*(4, 22) = 1.427, *p* > 0.05.

Recorded EEG data during the Nav, Enc, and Ret phases was analysed using Graph Theoretical analysis. The three segments of EEG data (prior to the task, just after cue presentation, and middle of the task) for each phase were processed separately and subsequently graph parameters were computed and analysed for the good and bad navigators.

### Theta band analysis

**Fig. 2:**
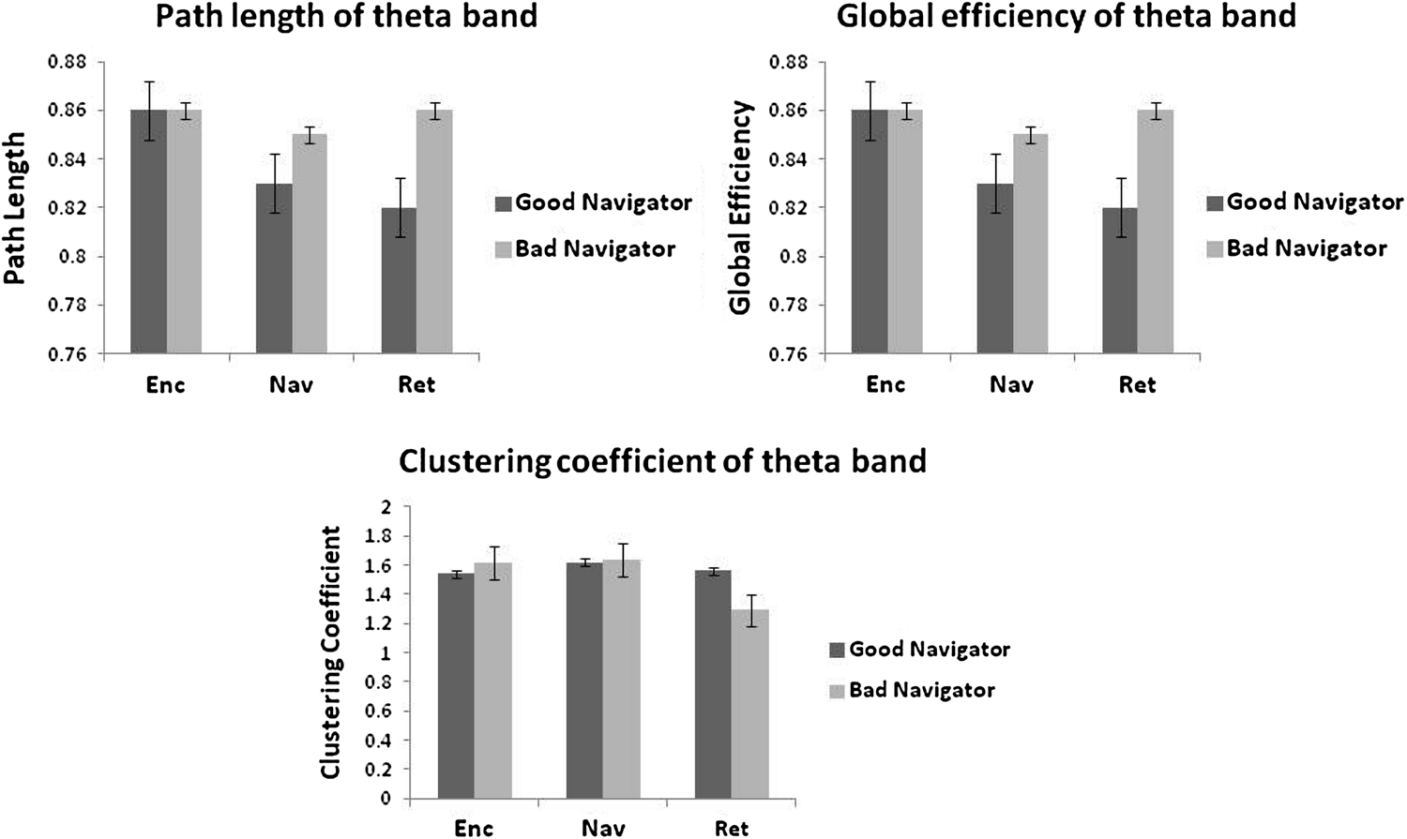
Graph parameters for Theta (4–7 Hz) wave synchronisation during encoding (Enc) phase, navigation (Nav) phase and retrieval (Ret) phase of the task. Only clustering coefficient (CC) was found to be significant in Ret phase, *p* = 0.001

The functional connectivity network of the bad navigators demonstrated higher values of PL, GE, and CC for the Enc and Ret phases compared to the Nav phase (Fig. 2).A reverse trend was observed in the Nav phase with network parameters in the good navigators where the values for PL, GE, and CC were found to be higher. During the Nav phase, CC was found to be significantly lower in the good navigator compared to the bad navigator, *F*(1, 84) = 10.86, *p* < 0.01.

### Alpha band analysis

**Fig. 3:**
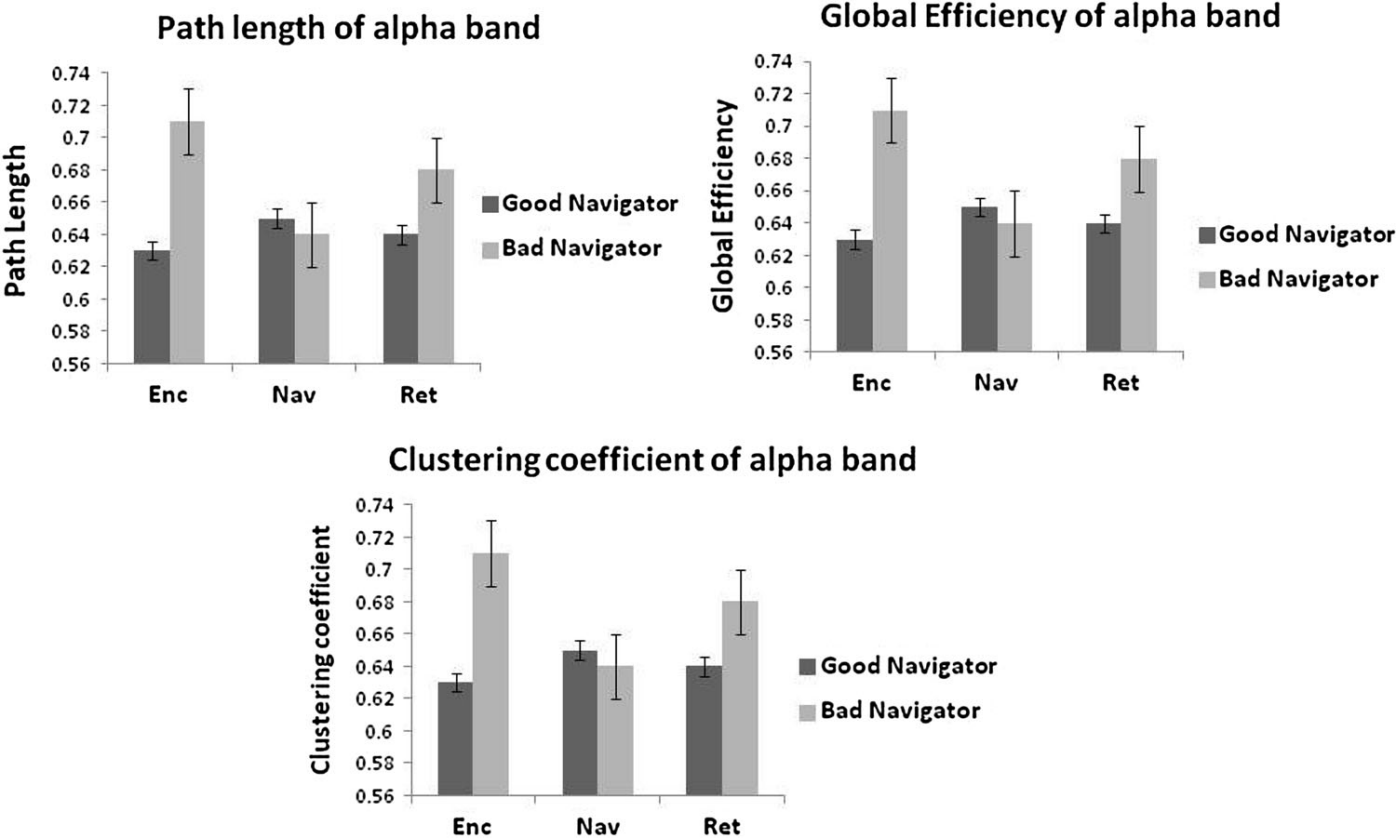
Graph parameters for Alpha (8–13 Hz) wave synchronisation during encoding (Enc) phase, navigation (Nav) phase and retrieval (Ret) phase of the task. Only clustering coefficient (CC) was found to be significant in Ret phase, *p* = 0.007

In the case of the bad navigators, alpha band analysis indicated higher values for PL, GE, and CC for all the three phases except the Nav phase. While for the good navigator, values of PL, GE, and CC were elevated in the Nav phase. (Fig. 3). It was noticed that the CC in the Nav phase for the bad navigators was significantly lower compared to the good navigator, *F*(1, 84) = 9.0, *p*$ < $0.01. As a consequence, the small world index (SWi) was computed in the Nav phase. Based on the participant’s recall scores, a scatter plot was drawn between the recall score and the network density for the Enc and Ret phases while for the Nav phase, only SWi was considered. The results were plotted only for alpha band, as a balance between inhibition and active processing could be achieved which would be easily correlated with functional connectivity (based on our assumption). Summarised graph parameters for all the three phases in the good and bad navigators are shown in Table 1 for theta and alpha bands.

**Table 1:** Mean (SD) for the graph parameters in different phases for theta and alpha bands

EEG bands	Phases	Graph parameters					
		Path length (PL)		Global efficiency (GE)		Clustering coefficient (CC)	
		Good navigator	Bad navigator	Good navigator	Bad navigator	Good navigator	Bad navigator
Theta	Enc	0.86 (0.04)	0.86 (0.08)	0.86 (0.14)	0.86 (0.14)	1.5 (0.14)	1.6 (0.14)
	Nav	0.83 (0.35)	0.85 (0.25)	0.83 (0.47)	0.85 (0.23)	1.6 (0.8)	1.62 (0.5)
	Ret	0.82 (0.14)	0.86 (0.15)	0.82 (0.44)	0.86 (0.71)	1.5 (0.14)	1.3 (0.14)
Alpha	Enc	0.63 (0.21)	0.71 (0.4)	0.63 (0.44)	0.71 (0.21)	0.63 (0.24)	0.71 (0.22)
	Nav	0.65 (0.01)	0.64 (0.02)	0.65 (0.12)	0.64 (0.22)	0.65 (0.41)	0.64 (0.56)
	Ret	0.64 (0.04)	0.68 (0.04)	0.64 (0.45)	0.68 (0.01)	0.64 (0.44)	0.68 (0.38)

We employed linear regression to test if the network density of the individual network could significantly predict recall scores (behavioural outcome). Linear regression was performed by regressing each participant’s performance against the total percentage connectivity of the individual network. Percentage connectivity was defined as the actual number of edges in the graph as a proportion of the total number of possible edges. The percentage connectivity was positively correlated with the recall score in both the groups for the Enc phase and Ret phase.

**Fig. 4:**
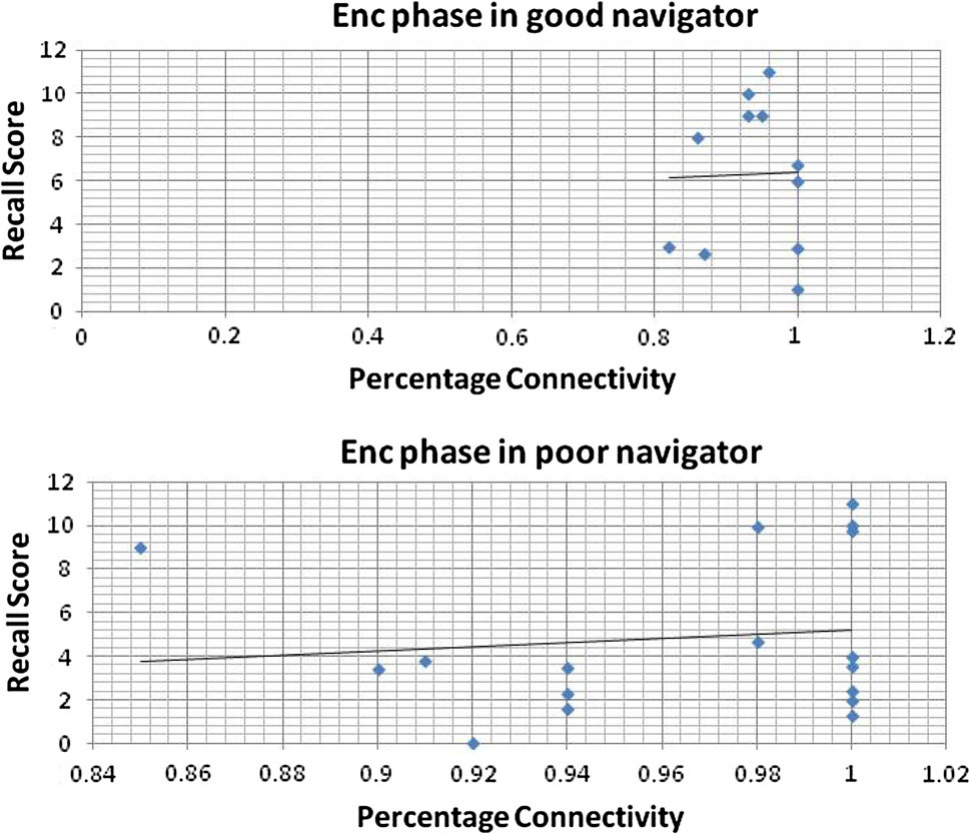
Scatter plot between recall score and total percentage connectivity during Encoding phase in good navigator (*R* ^2^ = 8%), and poor navigator (*R* ^2^ = 2%)

**Fig. 5:**
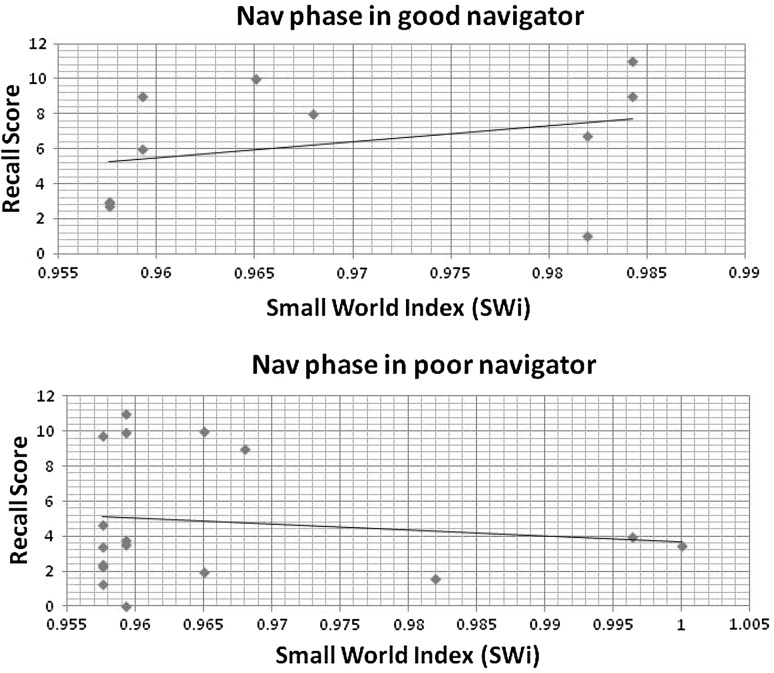
Scatter plot between recall score and small world index (SWi) during Navigation phase in good navigator (*R* ^2^ = 1%), and poor navigator (*R* ^2^ = 0.08%)

**Fig. 6:**
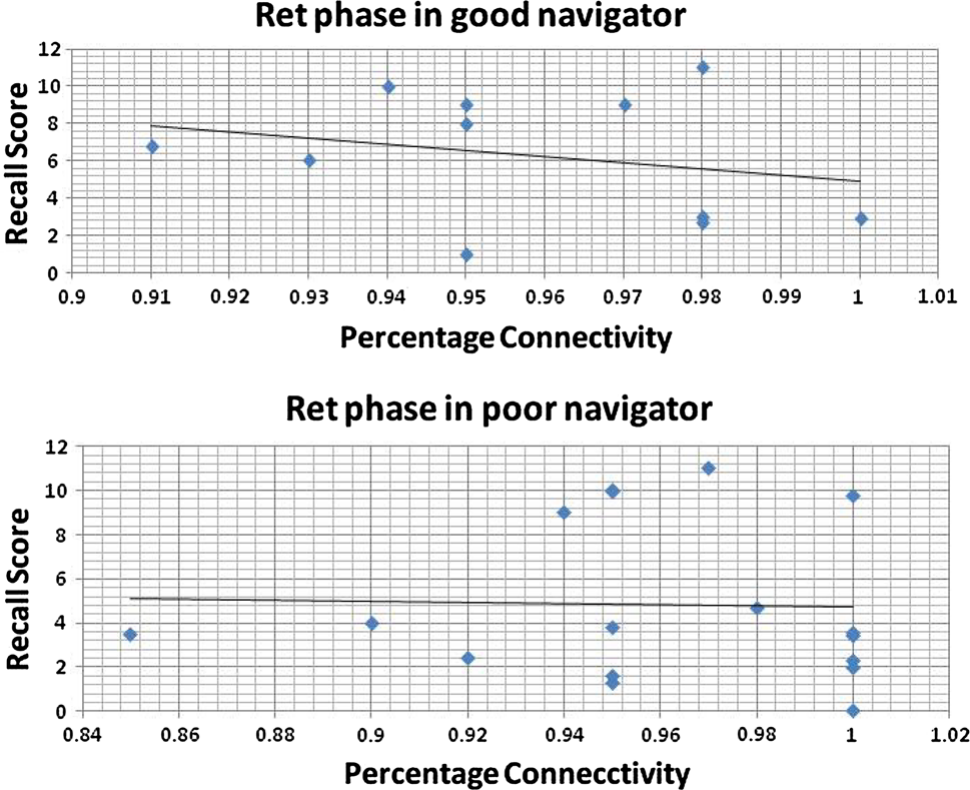
Scatter plot between recall score and total percentage connectivity during Retrieval phase in good navigator (*R* ^2^ = 2%), and poor navigator (*R* ^2^ = 5%)

However, the range of distribution was greater in the bad navigators for the Enc phase compared to the good navigator (Fig. 4). Differences were more visible in the Nav phase, where SWi was negatively correlated with recall scores in the bad navigators (Fig. 5). The scatter plot for the Ret phase is displayed in Fig. 6.

### Hub analysis

Hubs play a significant role in the information integration. In the present study, number and location of active hubs were compared within the group for recall scores. For the good navigators, the best performers (higher recall score) had the left frontal, anterior frontal, frontocentral, occipital and, temporal leads as the hubs, while the worst performers had the left frontal, anterior frontal, frontocentral, and occipital electrode positions as the hubs. On the contrary, for the bad navigators, similar hubs were active in the best and worst performers; however, a number of active hubs were lesser compared to the good navigators. Those hubs were left frontal, anterior frontal, and frontocentral sites.

## Discussion

The primary goal of the present study was to analyse the changes in the brain connectivity in people with different navigation skills, as they acquired and recalled survey and route knowledge. In addition, we aimed at gauging the potential effects of the FOR on the navigation performance applying Graph Theoretical  technique as an aid to identifying the connectivity patterns in participants with the good and bad navigation skills. No effect of strategy was found on the performance as revealed by non-significant effect of FOR on performance and response latency from online proclivity test. However, there was uneven performance distribution of participants (25% of best performers in the good navigator group while 45% in the bad navigator group), indicating navigation performance was independent of the spatial memory. The experiment was divided into three phases, Enc, Nav, and Ret, to evaluate encoding and retrieval stage separately. Graph Theoretical technique was applied on physiological signals to compute graph measures and network density/SWi for three phases separately. The insights and answers to the four main questions addressed by this study are the following: (1) the divergence in the brain connectivity was analysed with respect to the two main stages of spatial knowledge, Acquisition Stage (Enc and Nav phases) and Recall Stage (Ret phase) and it was found that the PL, GE, and CC varied across the phases in both the group; (2) our study indicated that the brain dynamics of the good navigators could be distinguished from those of the bad navigators on the basis of the CC value of the resultant functional connectivity network formed during the Nav phase; (3) there was no significant effect of FOR on the performance; (4) the differences in navigation skills had no bearing on the encoding of the spatial information or navigation skill was not necessarily accompanied by a different mechanism for the spatial information representation during the Enc phase.

Graph measures, GE, PL, and CC, were calculated for theta and alpha band. Only navigation phase in alpha band showed reduced values of graph parameters for the bad navigators. In addition, in retrieval phase, CC was found to be lower in the bad navigator compared to the good navigator. A higher value of CC means that the network had more local functional interconnections, and thus was more efficient in terms of local information transfer [32].The longer PL in the bad navigators signified less efficient information transfer during the Enc phase compared to the Nav phase. In the Ret phase, CC was higher for the bad navigators, indicating a higher number of local interconnections. This might suggest that during the Nav phase, bad navigators demonstrated gravitation towards a small world network in both the theta and alpha bands. The formation of a small world network also alluded a balance between functional integration and functional segregation in the local cortical regions. An elevated GE was observed in the bad navigators during the Enc and Ret phases. GE refers to the efficiency of information transfer between the nodes through multiple paths. Increased GE and smaller PL signified a reduction in noise and an increment in synchrony between different cortical areas. To sum up, only clustering coefficient of the theta band was sufficient to distinguish local information exchange for the good and bad navigator during the navigation phase and retrieval phase. However path length and global efficiency also varied within the good and bad navigator, but these parameters were unable to draw line of differences. This concludes that significant change in the underlying cortical interconnections was local rather than global in estimating individual differences for navigation skills.

Till now, graph parameters provided the individual difference based on the performance in the virtual navigation task. In terms of relationship between memory and navigation performance, scatter plot provided emerging pattern for the good and bad navigators. Memory as depicted through recall score became storehouse of accumulated spatial knowledge in the Enc and Nav phases. Variations in the recall scores among the two groups were correlated with the network density and SWi. This lent a perspective of overall network connectivity for both the groups during all the three phases. Higher percentage connectivity exhibited a greater degree of functional interactions. The increase in SWi signified a more efficient information transfer and a lower associated rewiring cost. The percentage connectivity was positively correlated with the recall score in both the groups for the Enc phase while the reverse was observed in both the groups for the Ret phase. However, in the Enc phase, range of dispersion was higher for the bad navigators compared to the good navigators. Differences were more evident in the Nav phase where SWi was negatively correlated with the recall scores in the bad navigators. This observation could be attributed by a possibility that people with the good navigation skills inclined to remember their path right from the first time they have encountered the map/route of the novel environment while people with the bad navigation skills put greater mental efforts in accumulating primary knowledge and acquired them through experience. The other reason for such a notable differences in the Nav phase might be that the bad navigators struggled to develop spatial knowledge during the Enc phase and subsequently, to compensate the loss of construction, they accelerated the assimilation of knowledge by constructing the small world network during first-person exposure (Nav phase) to the environment. This, in turn, explained the larger range of dispersion during the Enc phase in the bad navigators compared to the good navigators, whereas similar relations were established during the Ret phase in both the groups. To summarise, bad navigators displayed imprecise navigation skills reflecting the reduced connectivity in encoding spatial layout (knowledge acquisition through map and experience). When comparing Ret phase, percentage connectivity of both the groups had similar linear correlations with their recall score. This indicated that lesser brain resources were utilised by both the group in the Ret phase compared to the Enc phase, exhibiting lesser mental effort while recalling the spatial information [33]. However, the steep of curve was more sharper in the good navigator, demonstrating efficient means to operate in the limited resources. In conclusion, the differences between the bad and good navigators existed right from the beginning of the task. Bad navigators had more dispersion during the Acquisition stage, whereas Recall stage had the similar pattern as those of good navigators.

To distinguish the best and worst performers (on the basis of recall score) within the group, we used the node degree for estimating functional hubs. There were more functional hubs in the best performers having good navigation skills. More hubs indicated higher cortical synchronisation in the network [34]. There were fewer functional hubs for participants with best and worst recall scores with the bad navigation skills. Interestingly, participants having best recall scores resulted in numerous hubs and many more functional connections compared to participants having worst recall scores. An additional finding related to the hubs was the dominance of left hemispheric activation. According to hemispheric encoding/retrieval asymmetry (HERA) model [35], right-hemispheric activation is correlated with the episodic memory retrieval while left-hemispheric activation is correlated with the semantic memory retrieval. However, our findings were not congruent with the HERA model. One reason for this might be that the participants associated the episodic memory with the existing semantic maps of space to support their navigation in the given novel environment [36]. As proposed by Buzsaki and Moser [8], the integration of episodic memory with the navigation was supported by both episodic and semantic memory. Henceforth, memory interaction with the spatial knowledge embodied the formation of hubs in the left-hemisphere.

## Conclusions

We investigated the brain dynamics of subjects with the good and bad navigation skills using Graph Theoretical technique on low-density EEG data. This study also explored the potential role of a proclivity for distinct FOR on navigation performance. People with the good navigation skills showed more functional connectivity during the acquisition of spatial information (first-person perspective and aerial perspective) as compared to people with bad navigation skills. Additionally, people with bad navigational skills showed a negative correlation between the recall score and percent connectivity during navigation phase or during learning from a first-person perspective. Furthermore, both groups showed decrease in the functional connectivity during retrieval phase, demonstrating lesser recruitment of the resources as supported by fewer hubs activation. Moreover, the present study provided an interesting finding that information encoding in spatial memory was independent of the type of navigational skills demonstrated by an individual. Thus, the findings provide the variation in the underlying cortical activation associated with the individual's navigation skills. Similarly, extending towards the exploration of memory and FOR in the shaping of performance during navigation bestowed another dimension in understanding the role of other factors in the navigation skills. Although, this study explored the complex interplay of FOR and the perspective and their impact on the spatial information acquisition and recall, future studies should address the possible combined impact of different FOR and perspective pairs. Such knowledge would greatly refine our understanding of navigation at the neural level which can then be harnessed to design better training modules for personnel involved in military, transportation, forest, and rescue operations.
